# Transcriptomic and Physiological Analysis Reveal That α-Linolenic Acid Biosynthesis Responds to Early Chilling Tolerance in Pumpkin Rootstock Varieties

**DOI:** 10.3389/fpls.2021.669565

**Published:** 2021-04-23

**Authors:** Wenqian Liu, Ruoyan Zhang, Chenggang Xiang, Ruiyun Zhang, Qing Wang, Tao Wang, Xiaojun Li, Xiaohong Lu, Shunli Gao, Zixi Liu, Mengshuang Liu, Lihong Gao, Wenna Zhang

**Affiliations:** ^1^Beijing Key Laboratory of Growth and Developmental Regulation for Protected Vegetable Crops, China Agricultural University, Beijing, China; ^2^College of Life Science and Technology, HongHe University, Yunnan, China

**Keywords:** chilling, DEGs, pumpkin rootstocks, α-linolenic acid biosynthesis, multi-disciplinary aspect

## Abstract

Climate changes especially chilling stress affects cucurbit crops during winter seasonal production. Grafting to pumpkin rootstocks is widely used to improve the vigor of cucurbits, especially cucumber (*Cucumis sativus* L.) plants, in the face of chilling stress. In our study, multi-disciplinary aspect approaches were used to investigate growth changes of pumpkin under chilling stress. Firstly, the morphological and physiological characteristics of 14 pumpkin (*Cucurbita moschata*) varieties following different periods of chilling stress was analyzed by using physiological means. Mathematical results of principal component analysis (PCA) with chlorophyll-a, chlorophyll-b, carotenoid contents, chilling injury index and relative electrolyte permeability indicated that relative electrolyte permeability as the primary judgment index was best associated with the comparison of chilling tolerance in pumpkin rootstock varieties. Then, transcriptomic and DCMU (Diuron) application and chlorophyll fluorescence examination analysis of pumpkin leaves revealed that 390 *Cucurbita moschata* differentially expressed genes (CmoDEGs) that affect photosynthesis were upregulated in leaves. 127 CmoDEGs both in leaves and roots were enriched for genes involved in unsaturated fatty acid metabolism, suggesting that plasma membrane lipids are involved in chilling perception. The results of increased composition of unsaturated fatty acid in leaves and qRT-PCR analysis of relative mRNA abundance confirmed that α-linolenic acid biosynthesis was responding to pumpkin chilling tolerance. The integration of physiological, mathematical bioinformatical and biological analysis results contributes to our understanding of the molecular mechanisms underlying chilling tolerance and its improvement in cucumber grafted on pumpkin rootstocks. It provided an important theoretical basis and reference for further understanding on the impact of climate change on plant physiological changes.

## Introduction

Climate changes especially chilling stress is an abiotic stress that affects the growth and development of crops and leads to plant growth retardation or death (Taylor et al., 1974; Tester and Bacic, 2005). When a plant encounters chilling stress, a series of cellular responses are activated that allow the plant to adapt to the stress (Suzuki and Mittler, 2006; Kim and Tai, 2011; Maruyama et al., 2014). Cucumber (*Cucumis sativus* L.) is a major vegetable crop cultivated in winter, but it has a low tolerance for most types of stress, due to a shallow root system, which limits water and mineral uptake. Chilling stress from low temperatures (<10°C) that can limit the photosynthesis, then decrease the quality and productivity of cucumber fruit (Semeniuk et al., 1986). Grafting has been widely used in horticultural crop production, and cucumber tolerance to abiotic stress can be improved by grafting the plants onto suitable pumpkin (*Cucurbita moschata*) rootstocks (Zhu et al., 2008; Lee et al., 2010; Li et al., 2014; Xu et al., 2017). Rootstock resources benefit the agricultural and commercial properties of grafted horticultural plants in various ways. For cucumber, figleaf gourd (*Cucurbita ficifolia* Bouché) and pumpkin (*Cucurbita moschata*) rootstocks improve chilling tolerance and fruit production under cold conditions (Aslamarz et al., 2010; Xing et al., 2015; Niu et al., 2019).

Climate change causes a huge impact on the ecosystem, and the response of plants to climate change is mostly expressed by botanical signals. In cold environments, the plants plasma membrane is the primary site that perceives and responds to external cold signals (Lyons, 1973; Orvar et al., 2000), and membrane lipid composition influences membrane stability and the ability of the plant to adapt to cold stress (Zhu, 2016; Zhang et al., 2019). The activity of enzyme systems on the membrane decreases, the decomposition of organic matter reduces the degree of unsaturation of phospholipids and fatty acids within the membrane, and the plasma membrane changes from a liquid crystal state to a gel state. Ultimately, changes in the fluidity of the phospholipid membrane and the stability of the membrane lipid structure led to an imbalance in plant metabolism (Sangwan et al., 2002; Nakamura, 2017). Decreased membrane fluidity caused by cold especially affects the density of stomata, respiration (Grant and Loake, 2000; Aslani et al., 2009; Aslani and Vahdati, 2010), and destroys chloroplast ultrastructure and then inhibits the reactions of photosystem II (PSII), blocking thylakoid electron transport and chlorophyll biosynthesis, resulting in leaf chlorosis (Liu et al., 2012; Zhang et al., 2014).

The composition of the plasma membrane lipid system was constantly adjusted in response to the temperature changes of environment, so that the relative distribution of lipids in plasma membrane of different tolerant varieties will be different under chilling stress. Within the lipid membrane structure of cold-tolerant walnut genotypes, polyunsaturated fatty acids (PUFA), monounsaturated fatty acids (MUFA) and saturated fatty acids (SFA) constituted on average 63.8, 26.7, and 9.7% of fatty acid content, respectively (Sarikhani et al., 2021). Proline content played another important role in membrane protection as it acts like ROS scavenger in the chilling condition (Aslani et al., 2011). During unsaturated fatty acid formation process, the solidification of plasmomembrane occurs in FAD (Oleate Dehydrogenase)-deficient mutant *fad2* at higher temperature (18°C) than in *Arabidopsis thaliana* wild type (14°C) and in overexpressed *FAD3* lines (12°C) (Vaultier et al., 2006). This indicates that fatty acid desaturases (FADs) function in fatty acid desaturation during plant chilling stress and changes in fatty acid desaturase (FAD) activity during chilling stress result in alterations in cell membrane structure (Chinnusamy et al., 2007). Furthermore, the type of FADs affects the degree of fatty acid unsaturation of plant cell membrane lipids (Chi et al., 2011; Park et al., 2016; Tovuu et al., 2016). For example, *Arabidopsis FAD3*, *FAD7*, and *FAD8* influence the synthesis of C18 and C16 triene fatty acids in low-temperature conditions (Routaboul et al., 2000). *FAD7* and *FAD8* redundantly catalyze the transformation from 18:2 to 18:3, *FAD7* is involved in plant growth and development at room temperature, particularly *FAD8* is involved in plant responses to low temperature (Nair et al., 2009; Chen et al., 2015; Roman et al., 2015).

α-linolenic acid (ALA; C18:3) is an important unsaturated fatty acid, which accelerates the synthesis of linolenic acid and released from chloroplast galactolipids for JA biosynthesis (Blée, 2002; Li et al., 2002, 2020; Upchurch, 2008; Liu et al., 2019; Shi et al., 2019; Wasternack and Hause, 2013). α-linolenic acid helps maintain membrane integrity under low-temperature stress (Song et al., 2019) and activates JA-induced cold defense gene expression (Hu et al., 2013). Lipoxygenases (LOXs) are reported to catalyze the oxidation of fatty acids to oxidized lipids and to participate in responses to chilling stress (Upadhyay et al., 2019). 13(*S*)-Hydroperoxy-octadecatrienoic acid is a substrate for several enzymes, including terminal propylene oxide synthase (AOS), which affects JA biosynthesis by catalyzing 13-LOX (Porta and Rocha-Sosa, 2002; Schaller and Stintzi, 2009); 13-LOX, in turn, alleviates low-temperature-induced plant growth stress.

As different pumpkin rootstock varieties display variation in chilling tolerance (Huang et al., 2012), we set out to investigate the causes for these differences. Multi-disciplinary approaches were often used to investigate the plant growth changes and biological responses under climate changes, for example, transcriptomic reveal that the transcriptional regulation of plants responding to temperature changes (Vimont et al., 2020). Firstly, we analyzed and compared the morphological and physiological characteristics of 14 pumpkin varieties in response to different periods of chilling stress. To clarify further the early chilling response signals induced in pumpkin leaves, we identified differentially expressed genes (DEGs) in pumpkin leaves and roots in response to a 6-h chilling (14°C) stress by transcriptome analysis. Enrichment analysis of Gene Ontology (GO) and Kyoto Encyclopedia of Genes and Genomes (KEGG) categories within the DEG functional clusters indicated that unsaturated fatty acid metabolism pathway was induced during early chilling stress. These observations provide a theoretical basis for further examining the mechanism whereby pumpkin rootstocks improve the cold tolerance of grafted cucumbers.

## Materials and Methods

### Plant Materials and Cultivation

As different pumpkin rootstock varieties display variation in chilling tolerance, we mainly focused on the differences between different pumpkin rootstock varieties to beneficial cucumber grafting in the further studies. Therefore, fourteen commercial pumpkin (*Cucurbita moschata*) rootstock cultivars used in this study were listed as follow: 1. *Figleaf gourd* (FG), 2. Jingxinzhen 4 (JX), 3. Hemei 3(HM), 4. Riben Xuesong (RBX), 5. Dazuo Taimu (DZ), 6. Jinxiu Taimu (JX), 7. Beinong Liangzhen (BN), 8. M51, 9. Riben Lishi (RBL), 10. Huangjinyan (HJ), 11. Huofenghuang (HF), 12. Lizhiyuan (LZ), 13. Yukai Zhenmu (YK), and 14. Qianglishi(QL). These commercial pumpkin rootstock seeds were directly bought by WZ under permission from online shop (www.taobao.com, Alibaba Group, China). The materials were formally identified by WL.

For cultivation, 100–200 seeds of each variety were soaked in warm, sterile water at 55°C for 20 min and then at room temperature (28°C) for 20 min. Seeds were then incubated in a dark chamber at 28°C overnight. Germinating seeds were sown in 72-hole seedling trays containing a mixture of peat:vermiculite:perlite (2:1:1, by vol.) and were cultivated in a growth chamber under normal growth conditions (relative humidity 70%; 16 h/8 h light/dark; temperature 28°C/18°C day/night; light intensity 190–600 μmol m^–2^ s^–1^).

### Chilling Treatment

When plants were growing in two-true-leaf stage, three replicates each of 100 seedlings of each variety were subjected to growth chamber with chilling condition (relative humidity 70%; 16 h/8 h light/dark; temperature 4°C/4°C day/night; light intensity 190–600 μmol m^–2^ s^–1^) for 12 days. Another 100 seedlings of each varieties in each of three biological replicates remained in normal growth conditions. Plants were observed to evaluate the symptoms of chilling injury of different cultivars at the time points of 0, 3 days. The first true leaves from ten plants from each biological replicate were randomly harvested to measure the contents of chlorophyll-a, chlorophyll-b, and carotene and for relative electrolyte permeability analysis to evaluate chilling tolerance.

For RNA-seq analysis, pumpkin rootstock of the cultivar “*Qianglishi*” was divided into two groups at the two-true-leaf stage (10 seedlings per group). One group was subjected to chilling (relative humidity: 70%; 16 h/8 h light/dark; 4°C/4°C day/night; light intensity: 190–600 μmol m^–2^ s^–1^) for 6 h. A control group was grown in normal conditions (relative humidity: 70%; 16 h/8 h light/dark; 28°C/18°C day/night; light intensity: 190–600 μmol m^–2^ s^–1^). The first leaf of all 10 plants in each group were divided into three replicates and immediately frozen in liquid nitrogen and stored at −80°C until further analysis. Leaf samples from three randomly selected biological replicates from the control and chilled groups were sent for RNA-seq analysis (Oe-biotech, Shanghai) and quantitative real time PCR (qRT-PCR) analysis respectively. For qRT-PCR assay, the first leaf and root of pumpkin were sampled at 0h, 6h and 3d under chilling treatment.

### Evaluation of Chilling Injury Symptoms

Chilling injury symptoms of the pumpkin rootstock seedlings were evaluated after 3 d of chilling treatment using the Chilling Injury Index classification developed as previous report (Semeniuk et al., 1986).

Grade 0: No symptoms of injury.
Grade 1: The edge of first leaves is yellow or slightly dehydrated.
Grade 2: Dehydration spots appear in a small part of the first leaves; the other leaves are slightly dehydrated.
Grade 3: Dehydration spots appear in half of the first leaves; the other leaves are slightly dehydrated.
Grade 4: Dehydration spots appear in most of the leaf area; half of the other leaves are dehydrated.
Grade 5: Almost all the leaves are seriously dehydrated and wilted.

Chilling Injury Index (CII) = (1 × S1 + 2 × S2 + 3 × S3 + 4 × S4 + 5 × S5)/(*N*× 5), where S1–S5 are the numbers of plants with grade 5 symptoms and *N* is the total number of plants.

### Quantitative Analysis of Chlorophyll-a, Chlorophyll-b, and Carotenoid Content

The content of chlorophyll-a, chlorophyll-b and carotenoids was determined according to previous protocol (Li, 2000). In detail, the first true leaf was excised from nine plants with similar growth from each variety, dust was removed from the leaf surface with a brush and a hole was punched in the same area of each leaf with a punch, avoiding the main veins. Chlorophyll was extracted with 95% ethanol. After 24 h, the absorption value of the extract at the three wavelengths 649, 665, and 470 nm was determined with an ultraviolet spectrophotometer, and the content of each pigment in the extract was calculated using the formulae: Chlorophyl-a (Chla) = 13.95 × D665 − 6.88 × D649 (mg L^–1^); chlorophyll-b (Chlb) = 24.96 × D649 − 7.32 × D665 (mg L^–1^); carotenoids = (1,000 × D470 − 2.05 × Chla − 114.8 × Chlb)/245 (mg L^–1^).

### Relative Electrolyte Permeability Analysis

The relative electrolyte permeability was determined as previous reported (Dionisio-Sese and Tobita, 1998) with modifications. A total of 0.1 g leaf blade was bored with a punch and placed in distilled water of 10 mK at 28°C. The electrical conductivity of the distilled water was measured and defined as EC_0_. Following shaking for 2.5 h, the electrical conductivity of the leaf solution was determined and recorded as EC_1_. The leaf solution was incubated in water bath at 95°C for 0.5 h and the electrical conductivity of leaf solution was measured after cooling to room temperature, and the value assigned as EC_2_. The relative electrolyte permeability was determined as (EC_1_ − EC_0_)/(EC_2_ − EC_0_) × 100%.

### Principal Component Analysis (PCA)

The low-temperature tolerance of the 14 pumpkin rootstocks was compared and evaluated based on the chlorophyll-a, chlorophyll-b and carotenoid contents, the relative electrolyte permeability per unit area and the CII at 4°C for 3 d. The values and contribution of each component were analyzed by principal component analysis (PCA), which was conducted as previous described (Zainal et al., 2019).

The Component matrix^a^ was obtained by SPSS. By dividing the value of the component matrix corresponding to the principal component by the square root of the corresponding eigenvalue, the corresponding coefficient of each index in the two principal components was obtained; i.e., the coefficient = the value of the component matrix/sqtr (eigenvalue). The chilling tolerance indices were marked successively as X1, X2, X3, X4, and X5, and the standardized data were designated as ZXi. The obtained coefficient was multiplied by the corresponding standardized data to obtain the main component score:

(1) F1=0.278×ZX1+0.268×ZX2+0.221×ZX3+0.219×ZX4-0.235×ZX5.

F2=-0.159×ZX1+0.358×ZX2-0.647×ZX3+0.672×ZX4+0.238×ZX5.

The proportion of the corresponding eigenvalues of each principal component to the sum of the total eigenvalues of the extracted principal component was used as the weight with which to calculate the principal component synthesis model, and the following synthesis model was obtained:

F=0.183×ZX1+0.288×ZX2+0.032×ZX3+0.318×ZX4-0.132×ZX5.

### RNA Library Construction and Sequencing

At least 10 seedlings from each treatment group (control and chilling) were pooled into three replicates. The extracted RNA from the pooled first leaves and roots (>10 μg, concentration 1–2 μg μL^–1^) was subjected to RNA-seq. The RNA-seq libraries were generated using the NEBNext^®^ Ultra^TM^ RNA Library Prep Kit for Illumina^®^ (NEB, United States) following the manufacturer’s recommendations. The mRNA was purified from total RNA using poly-T oligo-linked magnetic beads. Purification fragmentation buffer was added to cleave the mRNA molecules into short fragments. First-strand cDNA was synthesized using random hexamer primers and M-MLV reverse transcriptase (RNase H). Second-strand cDNA synthesis was subsequently performed using DNA polymerase I and RNase H. NEBNext Adaptors with hairpin loop structures were ligated for hybridization. The resulting cDNA library was sequenced on an Illumina HiSeq2500 platform (Oe-biotech, Shanghai) to obtain paired-end reads with a length of 150 bp. Library quality was tested using the Agilent Bioanalyzer (Life Technologies, Carlsbad, CA, United States) 2100 system, and the genome reference was the *Cucurbita moschata* genome (http://cucurbitgenomics.org/). The original data set was deposited in the NCBI Small Read Archive (accession no. PRJNA552914).

### Analysis of Differentially Expressed Genes (DEGs)

The abundance of each gene was normalized and calculated via the Fragments Per Kilobase of exon model per Million mapped fragments (FPKM) method as follows (Mortazavi et al., 2008):

$$\text{FPKM}=\frac{10^{6}C}{NL/10^{3}}$$

where *C* and *N* represent the counts of mapped reads uniquely aligned to a unigene and the sum of reads sequenced that were uniquely aligned to total unigenes, respectively, and *L* represents the sum of a unigene in base pairs.

The NOISeq method was then used to identify DEGs between the normal and drought-stress transcriptome libraries according to the following criteria: fold change ≥ 2 and divergence probability ≥ 0.8.

KEGG pathway and GO enrichment analysis were both performed with the OmicShare tools (http://www.omicshare.com/tools). The gene ID lists of CmoDEGs in leaf and root under both normal and chilling condition used for KEGG analysis. The reference file with KEGG IDs of pumpkin were produced as the kopath type files in Gene Denodo Company (https://www.omicshare.com/tools/Home/Soft/kegg_anno) using pumpkin genes obtained from the *Cucurbita moschata* (Rifu) Genome (http://cucurbitgenomics.org/).

### RNA Extraction, cDNA Synthesis and Quantitative Real-Time PCR Assays of Genes

Total RNA of all pumpkin samples was extracted by using Rapid Extraction Plant Total RNA Kit (Huayueyang Biotech, Co., Beijing, China Beijing Huayueyang Biotechnology Co., Ltd. Cat#0416-50) according to the manufacturer’s instructions. To determine RNA quality and concentration, 1 μL of each RNA sample was analyzed by agarose gel electrophoresis (2%, agarose, 1× TBE) and quantified using a NanoDrop ND-2000 (Thermo Scientific, Waltham, MA, United States).

Reverse transcription of 1 μg RNA was performed using PrimeScript^TM^ RT kit (perfect real-time) (Cat#RR420A, Takara Biomedical Technology Co., Beijing, China) with gDNA eraser following the manufacturer’s instructions. qRT-PCR was performed in the QuantStudio^TM^ 6 Flex real-time PCR system (Applied Biosystems, CA Foster City, CA) with 40 cycles in accordance with the instructions of TB Green^®^ Premix Ex Taq^TM^ II (Tli RNaseH Plus) (RR820A, Beijing, Takara). The ΔΔCt method was used to normalize the values of the samples and *ACTIN*. The primer sequences used in this experiment are shown in Supplementary Table S1.

### Measurement of Fatty Acid Composition

Either after chilling treatment or at the same stage in non-chilled control plants, the whole first true leaves with petioles were harvested. Three replicates including three individual plants from each time point were sampled and stored in a −80°C freezer.

Frozen spouts of all samples (100 mg) were suspended in 100 μl of isopropanol/acetonitrile (1:1, v/v) and 5 μl internal standard of FFA. Then centrifuged at 2,500 *g* for 15 min at 4°C. Finally, the supernatant solution was centrifuged at 12,000 *g* for 10 min at 4°C and used for metabolomics analysis. As stationary phase, an ACQUITY UPLC BEH C8 2.1 × 100 mm, 1.7 μm column (Waters, Milford, MA, United States) was used suited for lipids retention. The mobile phase consisted of solvent A (acetonitrile/water (1:10 v/v)) and solvent B (isopropanol/acetonitrile (1:1 v/v)) with gradient elution. The flow rate of the mobile phase was 0.30 mL/min. The column temperature was maintained at 55°C. The injection volume is 3 μl.

### DCMU Application, Chlorophyll Fluorescence Imaging

The effect of DCMU application on pumpkin leaves was investigated by using chlorophyll fluorescence (*Fv/Fm*) under different temperature conditions. The first true leaves in two-leaf-stage pumpkin seedlings were sprayed with 100 μmol/L DCMU (Diuron, CAS#330-54-1, Aladdin Biochemical Technology Co., Shanghai) under normal and chilling stress.

Three pumpkin seedlings at different time points were placed in the dark for 30 min, and then scan and photograph the first true leaves of pumpkin using the Chlorophyll Fluorescence Imager (CF imager-CF0077, TECHNOLOGICA Co, British).

## Results

### Pumpkin Rootstock Enhances Chilling Tolerance in Cucumber Shoots

**FIGURE 1:**
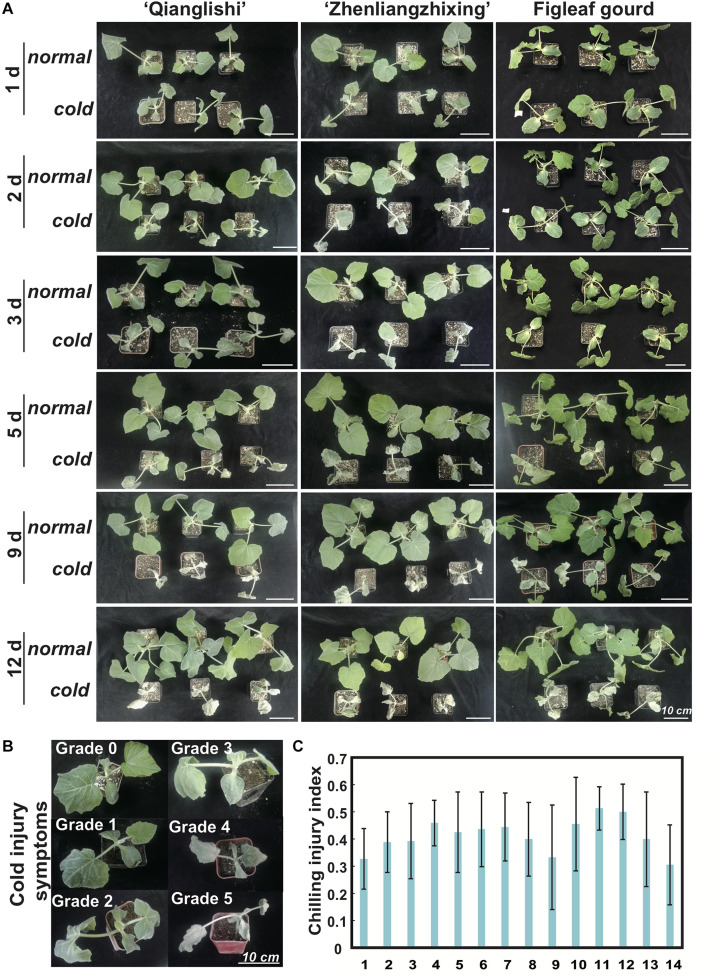
Seriousness of cold-injury symptoms vary between pumpkin rootstock varieties. **(A)** Phenotypic changes in three selected pumpkin rootstocks, “*Qianglishi*,” “*Zhenliangzhixing*,” and *figleaf gourd*, after different periods of chilling (4°C). Chilling treatments were applied to 100 seedlings in each of three biological replicates at the two-true-leaf stage. Another 100 seedlings in each of three biological replicates were kept in normal growth conditions. Symptoms were observed every day to evaluate the chilling injury of different varieties. **(B)** Chilling injury symptoms of pumpkin rootstock seedlings “*Qianglishi*” were evaluated using the chilling injury index (CII) classification (Semeniuk et al., 1986) after 3 d of chilling. Grade 0: no symptoms of injury. Grade 1: the edges of first leaves are yellow or slightly dehydrated. Grade 2: dehydration spots appear in a small part of the first leaves; the other leaves are slightly dehydrated. Grade 3: dehydration spots appear in half of the first leaves; the other leaves are slightly dehydrated. Grade 4: dehydration spots appear in most of the leaf area; half of the other leaves are dehydrated. Grade 5: almost all leaves are seriously dehydrated and wilted. **(C)** CII statistics in 14 pumpkin rootstock varieties. The detailed information of different numbers representing different pumpkin commercial varieties was listed in Material and Method part. Error bars indicate SD.

To select the rootstocks with the strongest chilling tolerance, we monitored the chilling injury symptoms of 14 pumpkin varieties daily. On the first and second day of chilling treatment, the edge of the first true leaves of most cultivars showed slight dehydration with curling in leaf edges, in contrast to those of plants grown under normal growth conditions. The rootstock *figleaf gourd* showed the greatest tolerance, without dehydration (Figure 1A). On the third day of chilling stress, most rootstock varieties began to dehydrate, and half of the true leaves in the cultivars “*Qianglishi*” and “*Zhenliangzhixing*” had wilted. On the fifth day of chilling stress, the true leaves and cotyledons of most rootstock varieties were dehydrated, but the edge of the first true leaf of the *figleaf gourd* had only begun to dehydrate (Supplementary Figure S1). We evaluated the plants using the chilling injury index on the third day of chilling stress, and the results indicated that *figleaf gourd* and “*Ribenxuesong*” were more tolerant than other rootstocks (Figures 1B,C).

**FIGURE 2:**
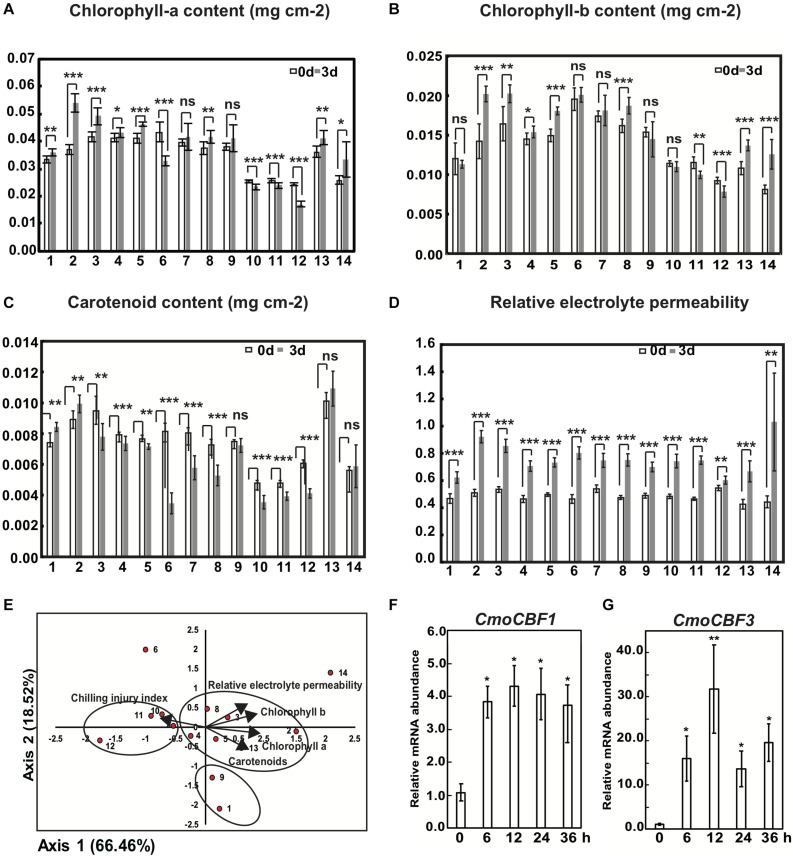
Physiological evaluation and principal component analysis (PCA) of 14 pumpkin rootstock varieties under cold treatment. The content of chlorophyll-a **(A)**, chlorophyll-b **(B)** and carotenoids **(C)** of 14 pumpkin seedlings after 3rd of chilling. **(D)** Statistics for relative electrolyte permeability (REP) of the first true leaves of seedlings from 14 pumpkin rootstock varieties after 3 d of chilling. **(E)** PCA for the content of chlorophyll-a, chlorophyll-b and carotenoids and REP and CII indices for 14 pumpkin rootstock varieties. Relative mRNA abundance of *CmoCBF1* **(F)** and *CmoCBF3* **(G)** in “*Qianglishi*” under chilling stress. Three replicates of the pooled first true leaves and 9–12 individual seedlings per each replicate were used for the analysis. Asterisks indicate highly significant differences compared with values from seedlings not exposed to chilling using the *t*-test for independent variables. The detailed information of different numbers representing different pumpkin commercial varieties was listed in Supplementary Table S1. Significance: ***p < 0.001; **0.001 < *p* < 0.05; *0.05 < *p* < 0.01; ns: *p* > 0.01. Error bars indicate SD.

Compared with the control, the fold changes in the chlorophyll-a, chlorophyll-b and carotenoid contents of the first true leaf of different rootstock varieties in response to chilling stress at 3 d differed. The increase in chlorophyll-a content of varieties 2, 3, and 5 was significantly higher than that in other varieties, and the decrease in the chlorophyll-a content of varieties 6, 10, 11, and 12 was significantly lower than that of the other varieties (Figure 2A). The increase in chlorophyll-b of varieties 2, 5, 8, 13, and 14 was significantly greater than that in other varieties, and the decrease in variety 12 was significantly lower than that in the others (Figure 2B). Chilling treatment caused a significant increase in carotenoid content in varieties 1 and 2 but a significant decrease in varieties 4, 6, 7, 8, 10, 11, and 12 (Figure 2C). According to the changes in chlorophyll-a and -b and carotenoid contents, variety 2 was chilling tolerant, whereas 12 was the variety most sensitive to chilling. To evaluate further the chilling tolerance of the 14 pumpkin rootstocks, we analyzed the relative electrolyte permeability, which reflects injury of the plasma membrane under chilling stress. Under chilling stress at 3 d, the relative electrolyte permeability of all 14 varieties except for variety 12 increased significantly (Figure 2D). This contrasts with the observed changes in chlorophyll-a and -b and carotenoid content.

We performed a PCA to integrate the relative changes in chlorophyll-a, chlorophyll-b, and carotenoid contents, the relative electrolyte permeability, and the chilling injury index as a means to evaluate the chilling stress tolerance of the 14 pumpkin varieties (Figure 2E and Supplementary Figure S2). Components 1 and 2 accounted for 66.46 and 84.97% of the total variation respectively (Supplementary Table S2), therefore PC1 and PC2 were extracted to evaluate the chilling tolerance of the 14 varieties. In the composition matrix table, chlorophyll-a and chlorophyll-b content had a higher load on PC1, which indicates that PC1 reflected the contribution of these two indicators. The load score of PC1 was positively correlated with the chlorophyll-a and chlorophyll-b contents (Supplementary Table S3). By contrast, carotenoid content and relative electrolyte permeability were higher in PC2 than the other components. Carotenoid content and relative electrolyte permeability were negatively and positively correlated with the score of PC2, respectively, so that a higher score of PC2 meant a lower chilling tolerance. The proportion of the corresponding eigenvalue of each principal component to the total eigenvalue of the extracted principal component was used as a weight with which to calculate the principal component comprehensive model. The weights of each index were in the order from biggest to smallest: relative electrolyte permeability, chlorophyll-b, chlorophyll-a,c hilling injury index, carotenoids. Therefore, relative electrolyte permeability, chlorophyll-a and chlorophyll-b could be used as key indices with which to screen for cucumber cold tolerance.

Taking the relative electrolyte permeability as the primary judgment index, that is, the smaller the relative electrolyte permeability score in the PC2 component, the stronger the cold tolerance of the corresponding pumpkin variety. The distribution of 14 varieties on PC1 and PC2 indicated that rootstocks 1 and 9 had the strongest chilling tolerance, and variety 6 was the rootstock most sensitive to chilling stress. Furthermore, no large differences in chilling tolerance were observed between varieties 1 and 9, among 7, 10, 11, and 12 or among 2, 3, 4, 5, 8, and 13. Thus, chilling injury symptoms and PCA of pumpkin seedling leaves indicated that the initial response of plants to chilling stress was leaf plasma membrane disorganization (Figure 2E and Supplementary Table S4).

In addition, we quantified the malondialdehyde (MDA) content using only varieties 1, 2, 3, 5, 8, 9, and 12, because other pumpkin varieties were affected by aphids at the seedling stage (Supplementary Figure S2A); therefore, PCA was performed with six indices and seven varieties. According to the principal component comprehensive model obtained after calculating the weights, the order of the weights for each index from biggest to smallest was: chlorophyll-b, relative electrolyte permeability, chlorophyll-a, MDA, cold damage index, chilling injury index. Therefore, relative electrolyte permeability, chlorophyll-b and chlorophyll-a can be used as key indicators with which to screen for the cold tolerance of cucumber. Chlorophyll-a and MDA had the highest load on PC1, but the PC1 score was negatively correlated with MDA, meaning that it was positively correlated to chilling tolerance. The load for chilling injury index and relative electrolyte permeability on PC2 was high; however, the chilling injury index and relative electrolyte permeability were negatively correlated with the chilling tolerance, which meant that PC2 was negatively correlated to chilling tolerance. Varieties 1 and 9 were more chilling tolerant and variety 12 was less tolerant than the other varieties. The integrated PCA of nine varieties on PC1 and PC2 indicated that rootstocks 1 and 9 were the most chilling tolerant and variety 12 was the most sensitive rootstock. Furthermore, we did not observe any large differences in chilling tolerance between varieties 1 and 9, or among varieties 2, 3, 5, and 8 (Supplementary Figure S2B).

The values for the chilling tolerance of the various pumpkin rootstocks differed when different physiological indices were considered for the evaluation. PCA with multiple indices determined that relative electrolyte permeability was most correlated with chilling tolerance and that variety 1 was the most tolerant and variety 12 was the most sensitive.

### Chilling-Induced CmoDEGs in Leaves and Roots Are Related to Unsaturated Fatty Acid Biosynthesis

**FIGURE 3:**
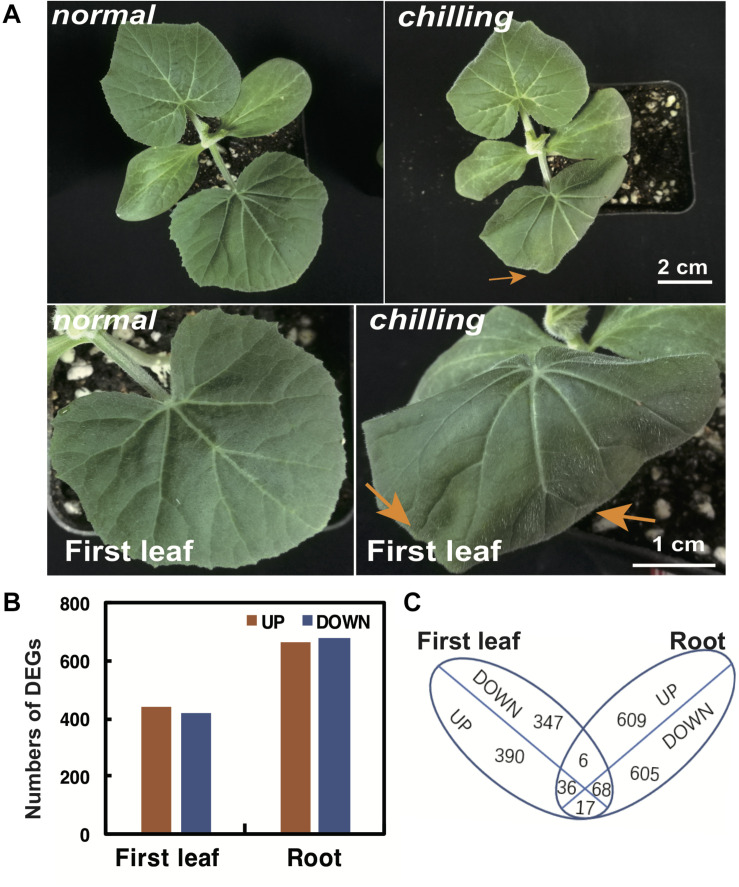
Analysis of differentially expressed genes (DEGs) between leaves and roots of pumpkin after 6 h of chilling. **(A)** Chilling injury symptoms of the leaves of pumpkin rootstock seedling after 6 h of chilling. Yellow arrows indicated wilted and wrinkled edge of first true leaves. **(B)** The number of up- and downregulated DEGs in the first leaves and roots of pumpkin compared with those in the 0 h normal condition. **(C)** Venn diagram of up- and downregulated DEGs in pumpkin first leaves and roots.

To elucidate the early responses to chilling in pumpkin leaves, we assessed chilling symptoms in the first two true leaves of the medium-tolerant variety “*Qianglishi*.” qRT-PCR revealed that the mRNA abundance of *CmoCBF1* and *CmoCBF3* was highly induced at 6 h chilling treatment (Figures 2F,G). At 6 h chilling treatment, the first true leaf seedling started to dehydrate at the edges but other leaves did not (Figure 3A). Therefore, we speculated that the first true leaf might be more sensitive to chilling, because it is the main source tissue with the largest area for photosynthetic assimilation. To address this hypothesis, we performed RNA sequencing (RNA-seq) with samples from the first true leaf and roots without chilling and after 6 h chilling. A total of 864 DEGs were identified in pumpkin leaves, 443 of which were upregulated and 421 were downregulated (Figure 3B). Out of the upregulated genes, 390 were exclusively upregulated in leaf tissue (Figure 3C and Supplementary Tables S5, S6).

**FIGURE 4:**
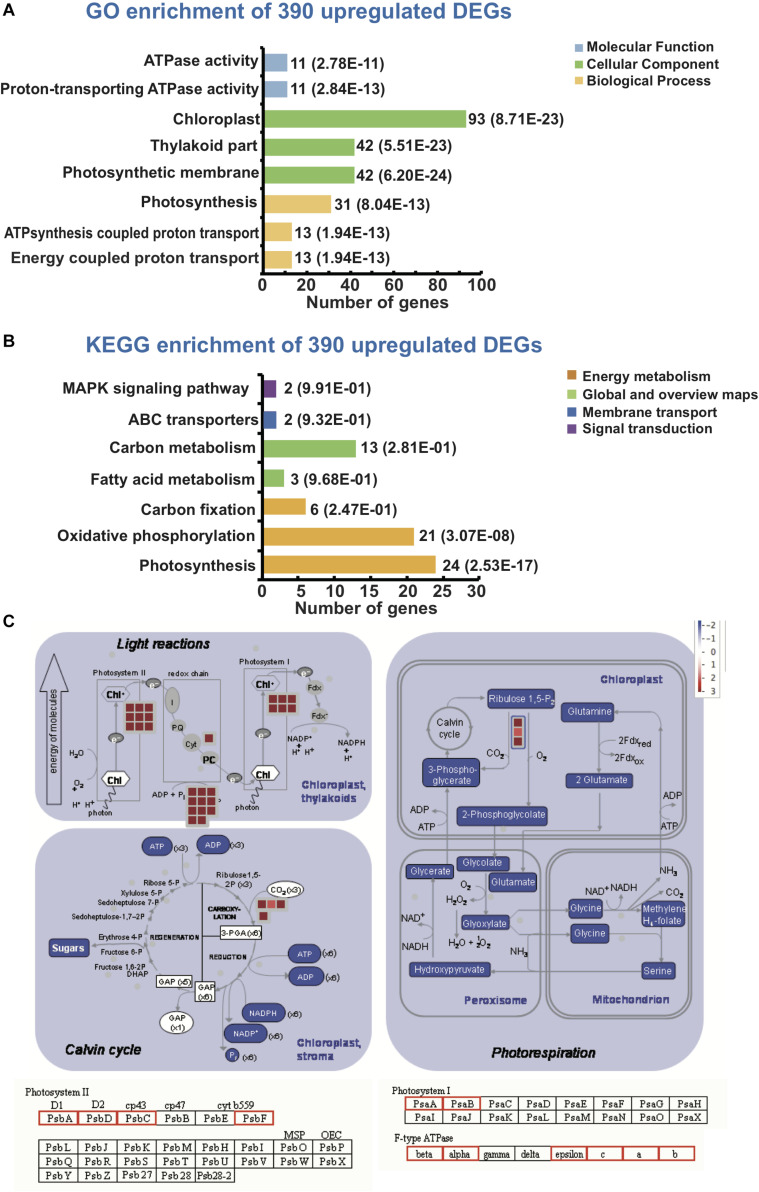
Gene Ontology (GO) enrichment and KEGG pathway annotation of 390 upregulated pumpkin DEGs in leaves. **(A)** GO enrichment and **(B)** KEGG pathway analysis of 390 upregulated pumpkin DEGs in leaves. Colored graphs indicate the classifications of molecular function, cellular component and biological process. The numbers beside each graph represent the number of genes in the cluster and the *P*-value. **(C)** Pathway of photosynthetic metabolism for the 390 upregulated pumpkin DEGs in leaves. The boxes indicate the various metabolites encoded by the upregulated DEGs in leaves after 6 h cold treatment compared with those in the 0 h treatment. The FIGURE was sourced from “Photosynthesis” in Mapman (MapManInst-3.5.1R2).

**FIGURE 5:**
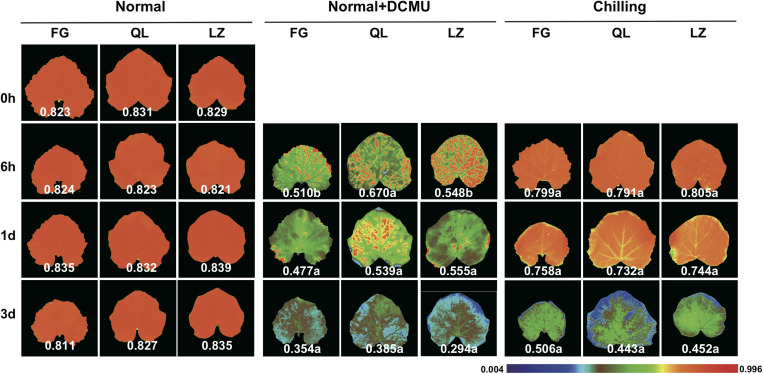
The effect of DCMU application on the chlorophyll fluorescence (*Fv*/*Fm*) of first leaf under different conditions. Three individual plants at each time points were used for analysis. Student *t*-test was used to analyzed the significant difference. Different letters indicate significant difference in each time points comparing with normal (*P* < 0.05).

GO classification and KEGG enrichment analysis was performed to determine the functions of the chilling-induced DEGs in leaf and root tissues. A total of 390 upregulated leaf-specific *Cucurbita moschata* DEGs (CmoDEGs) were enriched in photosynthesis and energy-coupled proton transport, chloroplast and plastid and ATPase activity categorized GO classifications (Figure 4A and Supplementary Table S7). They were enriched in KEGG pathways of fatty acids, carbon metabolism, ABC transporters and MAPK signaling, and were particularly enriched in the energy metabolism of carbon fixation, oxidative phosphorylation and photosynthesis pathways (Figure 4B and Supplementary Table S8). The 390 upregulated CmoDEGs also included genes involved in photosystems I and II, as well as a gene encoding an F-type ATPase involved in photosynthetic light reactions (Figure 4C). In order to explore the relationship between chilling damage with photosynthesis, we applied a photosynthetic inhibitor DCMU on the pumpkin first true leaves under different treatment. Chlorophyll fluorescence was used to measure the degree of chilling damage to leaves under different treatments. The results showed that that chilling injury does damage the photosynthesis of leaves, and “Qianglishi” leaves performed more tolerant to DCMU application (Figure 5). These data indicate that the majority of CmoDEGs that specifically respond to chilling stress in leaf tissues are involved in photosynthetic process, which suggests that chilling stress mainly affects leaf photosynthetic activity. Similarly, 620 upregulated CmoDEGs by chilling stress in the root were mainly responding to ethylene, oligopeptide transport, organ senescence and responding to temperature stimulus (Supplementary Table S9). The KEGG pathways associated with these 620 root-produced CmoDEGs included sulfur energy metabolism, carotenoid biosynthesis, lipids of α-linolenic acid metabolism, and tryptophan and phenylalanine metabolism pathways (Supplementary Table S10). The GO and KEGG enrichment analyses indicated that in roots, the chilling-specific CmoDEGs were mainly involved in oligopeptide transport, ethylene-induced senescence, signal transduction and oxidoreductase activity, which suggests that roots function as a source of signaling hormones that are then transported into the shoot.

**FIGURE 6:**
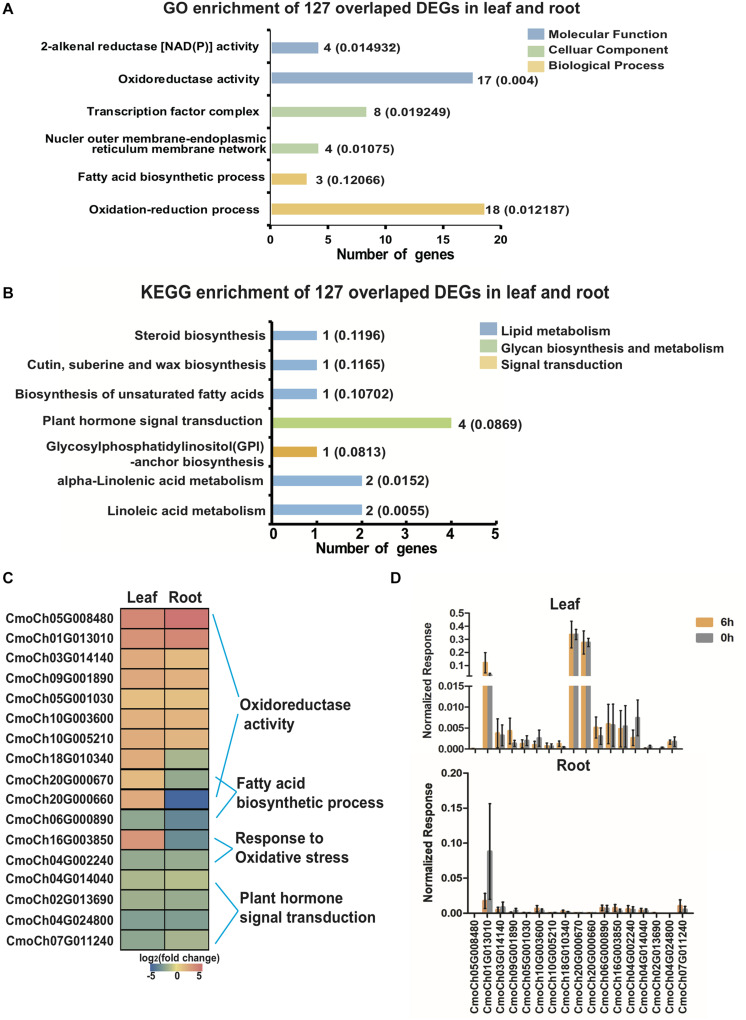
GO enrichment, KEGG pathway annotation and 127 selected overlapped CmoDEGs in leaves and roots. **(A)** GO enrichment and **(B)** KEGG pathway annotation of 127 overlapped CmoDEGs in leaves and roots. Colored graphs indicate the classifications of molecular function, cellular component and biological process. The numbers beside the graphs represent the number of genes in the cluster and the *p*-value. **(C)** Heat map of the top upregulated 14 DEGs in leaves and roots. These are enriched for oxidoreductase activity, fatty acid biosynthesis and responses to oxidative stress pathways. **(D)** The normalized FPKM level of 14 genes in leaf and root tissues. Each sample includes three replicates for the transcriptome data. Error bars indicate SD.

To further determine the similarities between the chilling-induced transcriptomes in leaf and root tissue, DEGs were found to be induced by chilling in leaves and roots. A total of 127 CmoDEGs were mainly enriched in the GO categories of cytoplasmic cellular components, and dioxygenase, oxidoreductase and catalytic activity of molecular function and also enriched in KEGG pathways of unsaturated fatty acid biosynthesis and α-linolenic acid pathways of lipid metabolism and in plant hormone transduction (Figures 6A,B and Supplementary Tables S11, S12). Among of them, seven genes were involved in oxidoreductase activity, and two involved in fatty acid biosynthesis that were specifically upregulated in leaves (Figure 6C). The normalized FPKM values for nine upregulated genes indicated that the mRNA abundance of CmoCh20G000660 and CmoCh20G000670 was highly upregulated in leaves, whereas that of CmoCh01G013010 was highly induced in both leaves and roots (Figure 6D). This indicates that chilling-induced CmoDEGs in leaves and roots are enriched for transcripts involved in lipid metabolism and oxygenase activity.

### Genes Involved in α-Linolenic Acid Metabolism Are Upregulated in Leaves During Early Chilling Stress

**FIGURE 7:**
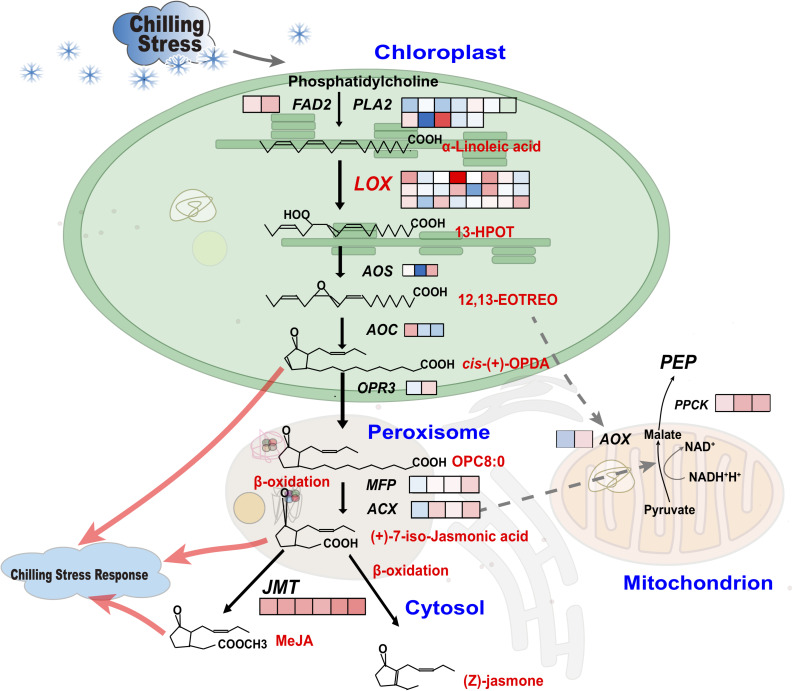
Unsaturated fatty acid, β-oxidation and energy metabolism pathways in leaves and roots of pumpkin rootstocks. Fatty acid desaturase and phospholipase genes regulate the production of α-linolenic acid in chloroplasts. Components of linolenic acid metabolism and subsequently unsaturated fatty acid metabolites of the α-linoleic acid metabolism pathway are metabolized by *LOX/AOS/AOC/OPR3*. The processes of OPC8:0 formation from fatty acid hydroperoxide, β-oxidation and MeJA biosynthesis occur in the peroxisome and are regulated by *MFP/ACX*. The *ACXs* are involved in the conversion of pyruvate to malate, which is associated with NAD energy production in mitochondria. In addition, *AOXs* mediate signal transduction between mitochondria and chloroplasts. Boxes indicate the log_2_ (fold change of FPKM) changes in gene expression levels. The different boxes indicate the corresponding homologous DEGs in pumpkin transcriptomic analysis between normal and chilling conditions.

Based on the GO enrichment and KEGG pathway analysis for 127 chilling-induced CmoDEGs in leaves and roots, we focused on those involved in lipid metabolism and oxidase activity. The CmoCh20G000660 and CmoCh20G000670 genes encode LOXs that regulate the conversion of α-linolenic acid to 13*S*-hydroperoxy-9*Z*,11*E*,15*Z*-octadecatrienoic acid (13-HPOT) and other unsaturated fatty acids to produce additional fatty acid hydroperoxides. CmoCh06G000890 encodes FAD2, which is a critical enzyme in α-linoleic acid biosynthesis, and CmoCh01G013010 encodes PEP, which is localized to the mitochondria and catalyzes 2-alkenal reductase [NAD(P)] activity to provide energy. CmoCh16G003850 encodes endonuclease or GH and is localized to the peroxisome, where it participates in chilling-induced oxidative stress (Figure 7 and Supplementary Table S13). The α-linoleic acid metabolic pathway involves multiple α-linoleic acid and unsaturated fatty acid biosynthesis pathways. The *PLA2* and *FAD2* are critical in α-linoleic acid biosynthesis, each of which regulates one node of the pathway. *DOX*s, *LOXs*, and *FADs* have key functions in the initial step of the parallel conversion of α-linolenic acid into other unsaturated fatty acids. In pumpkin α-linolenic acid metabolism, *DOX* regulates one metabolic node, and *LOX* catalyzes two metabolic nodes and the conversion of α-linolenic acid to fatty acid hydroperoxides. *HPL* and *AOS* regulate the metabolic flux of two metabolic nodes and the transformation of fatty acid hydroperoxides to form a variety of unsaturated fatty acids. Furthermore, *AOC* and *OPR* each regulate the biochemical reaction of one α-linolenic acid metabolic node, and *AOCX* and *MFP* regulate three metabolic nodes. We established that genes encoding those enzymes are involved in regulating the conversion of α-linolenic acid to JA during chilling responses (Figure 7).

**FIGURE 8:**
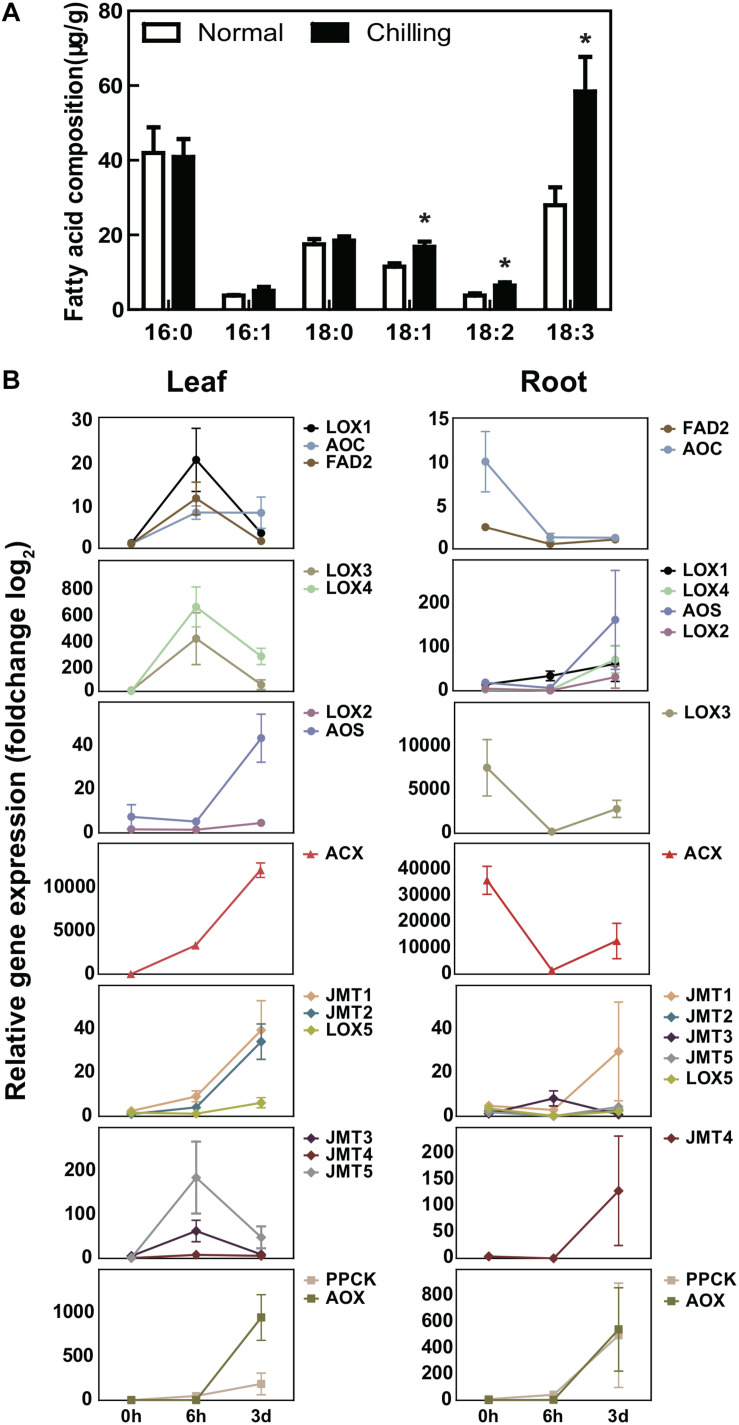
The relative mRNA abundance of unsaturated fatty acid pathway genes under chilling stress. **(A)** Changes in fatty acid composition of pumpkin leaves under normal condition and after 6 h of cold treatment. Fatty acids were measured by using pumpkin first leaf in three individual plants at each time points treatment. **(B)** The relative abundance of pathway genes involved in the synthesis of unsaturated fatty acids in pumpkin chloroplasts, peroxisomes and mitochondria are different in leaves and roots at 0, 6, 3 d chilling stress. *FAD2*, *LOXs*, and *JMTs* in leaves were first induced by chilling at 6 h and then deduced at 3 d chilling condition, but showed the opposite trend in roots. *AOS* showed a tendency to first decrease and then increase in both leaves and roots. *AOC* was up-regulated after chilling in leaves and down-regulated in roots. *PPCK* and *ACX* were both up-regulated in the leaves and roots. *ACX* was up-regulated in the leaves, and firstly down-regulated and then up-regulated in the roots. Different colors represented different genes. Each sample includes three replicates for the transcriptome data. Error bars indicate SD. Student *t*-test was used to analyzed the significant difference between mRNA abundance at 6 h, 3 d comparing with 0 h. *Indicates significant difference in (*P* < 0.05). Pumpkin *ACTIN* gene was used as the internal reference, and the graphics are generated by GraphPad Prism 5.0.

In order to verify our conjecture, we carried out fatty acid determinations on the first true leaves of pumpkin under normal conditions and low temperature environments (Figure 8A). The results showed that the content of unsaturated fatty acids in pumpkin increased significantly after chilling stress, especially oleic acid (18:1), linoleic acid (18:2), and linolenic acid (18:3) were significantly higher than normal conditions. Although other unsaturated fatty acids have also increased significantly but the overall content is low, such as eicosatrienoic acid (20:3) and docosamonoenoic acid (22:1) (Supplementary Table S14). Quantitative RT-PCR analysis on the first true leaves and roots of pumpkin seedlings was performed to test the genes in α-linolenic acid synthesis pathway responding to chilling stress at three time points. The results indicated that the abundance of *FAD2*, *LOXs*, *AOC*, *JMTs*, *PPCK*, and *ACX* in pumpkin leaves were both induced under 6 h and 3 d chilling treatment (Figure 8B). These results showed coordinate trend with RNA-Seq data, indicating the reliability of transcriptome profile analysis data (Figure 8B and Supplementary Table S13). qRT-PCR analysis also exhibited the abundance of *FAD2*, *LOXs*, *AOC*, *JMTs*, and *ACX* in roots were decreased at 6 h and then increased at 3 d chilling conditions, while the abundance of *PPCK* and *AOX* was continuously increased at both chilling time points, it indicated mitochondria mediated energy metabolism occurring in the roots of pumpkin was the continuous biological reaction responding to long-term chilling environment.

## Discussion

### Relative Electrolyte Permeability Is the Most Effective Indicator to Evaluate Cold Tolerance in Diversity of Crops

To evaluate the cold tolerance in different crop cultivars, three methods were compared: 1) thermal analysis; 2) evaluation of tissue health after controlled freezing; and 3) field observations (Huang et al., 2012). Normally electrolyte leakage/permeability and stomatal closure can be used to evaluate tissue health after controlled freezing (Niu et al., 2019). Firstly, chilling induces plant dehydration and increases ABA content resulting in a decrease of stomata density (Aslani and Vahdati, 2010). Then, the plasma membrane is composed of lipid bilayers and is important for plant biological responses. When plants are subjected to external cold stress, plasma membrane fluidity decreases and membrane lipids change from a liquid crystal state to a gel state, resulting in an increase in membrane permeability, relative electrolyte leakage and intracellular ion imbalance (Grant and Loake, 2000; Huang et al., 2015; Masouleh et al., 2019). MDA is also the product of cell membrane lipid peroxidation and can therefore be used to measure the level of membrane lipid peroxidation under stress (Supplementary Figure S2B).

Chilling stress significantly affects chloroplast ultrastructure and leads to a significant decrease in thylakoid accumulation, and the resulting loss of chlorophyll is accompanied by a decrease in photosynthetic efficiency (Wada et al., 1990; Routaboul et al., 2000). The data here show that pumpkin varieties with the highest chlorophyll content might have the best photosynthetic capacity and be most tolerant to chilling stress (Figure 2 and Supplementary Figure S2B). Although the evaluation of cold tolerance in pumpkin is complex, requiring the evaluation of several physiological and biochemical indices and extracellular and extracellular signals, as well as a comprehensive analysis of cold-stress genes expression and proteins. Here, we could conclude that the electrolyte leakage/permeability is the most effective indicator to evaluate cold tolerance in diversity of crops.

### Unsaturated Fatty Acid Biosynthesis Is Increased Both in Leaves and Roots of Pumpkin Rootstocks During Early Chilling Stress

Chilling causes the production of excessive SOD in leaves, which trigger a series of free-radical oxidation reactions on the double bonds of unsaturated fatty acids on the membrane, leading to cell membrane peroxidation. To maintain the morphology and structure of the plasma membrane, osmotic regulatory compounds, such as inorganic salts, proline, soluble sugars and soluble proteins, accumulate continuously in cold-tolerant varieties and regulate the fluidity of the biofilm to adapt to changes in temperature (Ruelland et al., 2009; Kishor and Sreenivasulu, 2014). In addition, stress is endured in chilling-tolerant pumpkin rootstocks through an increase in the content of unsaturated fatty acids or the scavenging of compounds such as ROS and SOD by the antioxidant enzyme system (Lyons, 1973; Niu et al., 2019). The involvement of these mechanisms in cold stress was demonstrated by the function of 127 upregulated DEGs in leaves and roots in membrane lipid and oxidoreductase pathways (Figures 6A–C).

The α-linolenic acid metabolism of unsaturated fatty acid biosynthesis was particularly enriched by chilling stress (Figure 6B). Since α-linolenic acid is produced by FAD2 (Li et al., 2003) and PLA2 from phosphatidylcholine (Figure 7), the metabolic flux of α-linoleic acid might represent the gateway to the biosynthesis of a variety of unsaturated fatty acids, and the reaction products of downstream genes such as *LOX*, *AOS*, *AOC*, and *OPR3* can transfer signals from the chloroplast to the peroxisome to affect the synthesis of JA (Upadhyay et al., 2019). The functions of the peroxisome include catalyzing the β-oxidation of fatty acids, decomposing VLCFAs into short-chain fatty acids under the regulation of MFP and ACX, regulating the formation of JA by α-linolenic acid during chilling stress, and affecting the biosynthesis of unsaturated fatty acids, thus protecting cells (Cao et al., 2009). JA and its derivatives also play an active role in regulating plant development and abiotic stress responses, because the abundance of α-linolenic acid, which is the substrate of JA biosynthesis (Wasternack and Hause, 2013).

Except for chloroplasts producing ROS (superoxide anions, hydrogen peroxide, hydroxyl radicals, and singlet oxygen), mitochondria and peroxisomes also produce ROS and many metabolites, which affect the expression of cold-responsive genes and cold tolerance (Dong et al., 2009; Mignolet-Spruyt et al., 2016). As part of the low-temperature response, the AOX mediates signal transduction between the mitochondria and chloroplasts and participates in the activity of 2-enal reductase [NAD(P)] to provide energy (Figures 4, 7). Additionally, it inhibits the formation of ROS, optimizes photosynthesis, affects the production of PEP and prevents cell over-reduction, thus facilitating environmental stress responses (Zhang et al., 2010; Yoshida et al., 2011; Dahal et al., 2014). The change in the AOX-derived plastid NADPH/NADP^+^ ratio and malic acid/oxaloacetic acid shuttle system might represent a metabolic signal that regulates plastid protein transport and chlorophyll synthesis. This is important for maintaining communication between the chloroplasts and mitochondria and for improving carbon assimilation and fixation via the TCA cycle.

The increasing abundance of *ACX* in the leaves is higher than that of other genes, indicating that the biological changes in leaves in responding to extreme temperature stimuli were mainly relating to ROS signaling in peroxisomes, thereby affecting the downstream β-oxidation and (Z)-jasmone production. Thus, these unsaturated fatty acids catalyzed by *JMT*s also promoted the biosynthesis of MeJA. The relative abundance of *FAD2*, *LOXs*, *AOC*, *JMTs*, and *ACX* in roots all decreased at 6 h and then increased at 3 d chilling condition, especially the foldchange of *ACX* abundance was larger than other genes (Figure 8B and Supplementary Table S12), suggesting that ROS signal transduction in peroxisomes play an important role in response to low temperature stress. Interestingly, the abundance of *PPCK* and *AOX* localizing in mitochondria were both increased with identical changes in leaves and roots, rising a possibility that *AOX* and *PPCK* in mitochondria participated in the production of PEP and prevented cell over-reduction, then regulated carbon assimilation and fixation via the TCA cycle. We conclude that the leaves and roots of pumpkin rootstocks acquire chilling tolerance by increasing the biosynthesis of unsaturated fatty acids in the chloroplasts, peroxisomes and mitochondria.

### Integrating of Multi-Disciplinary Aspects Revealed a Comprehensive Performance of Plant Responding to Climate Change

Plants will adapt to the external environment changes to the greatest extent by changing their own physiological morphology and biochemical responses (Suzuki and Mittler, 2006; Maruyama et al., 2014; Zhu, 2016). However, the physiological and molecular responses in whole plant are very complex so that it is challenging for us to figure out the mechanism of how and where the plant adaptation occurs. Therefore, multi-disciplinary approaches were often used to investigate the plant growth changes and biological responses under climate changes, for example, the integration of large-scaled RNA-seq data analysis and transcriptional investigation revealed that the transcriptional regulation of plants responding to temperature changes (Cortijo et al., 2017). In our study, the combined investigations on bioinformatic, mathematics, phenotypes, and physiological gained an effective result that can explain how and where pumpkin cold tolerance occurs (Figures 7, 8). It means that the multi-disciplinary approaches raised a possibility for the evaluation on the chilling tolerant crops varieties. Therefore, it is a hot spot and direction to study the gene function in controlling agricultural traits in the future by constructing a network relationship by combining plant physiological, plant biochemical and ecological characteristics when crops facing to climate change.

## Data Availability Statement

The datasets presented in this study can be found in online repositories. The names of the repository/repositories and accession number(s) can be found in the article/Supplementary Material.

## Ethics Statement

We declare that these experiments comply with the ethical standards and legislation in China, and all commercial pumpkin cultivars were collected in accordance with national and international guidelines.

## Author Contributions

LG and WZ conceived and designed the experiments. WL, RuiZ, XLi, TW, XLu, ZL, and ML performed the experiments. WL, RuoZ, and WZ wrote the manuscript. WL, RuoZ, CX, QW, and WZ analyzed the data. All authors read the final version of this manuscript and approved it for publication.

## Conflict of Interest

The authors declare that the research was conducted in the absence of any commercial or financial relationships that could be construed as a potential conflict of interest.

## Footnotes

a 2 components extracted.

## Supplementary Material

Supplementary Figure 1 Phenotypic changes in 14 pumpkin rootstocks after 5 d of chilling. Chilling treatments were applied to 100 seedlings at the two-true-leaf stage in each of three biological replicates, whereas another 100 seedlings in each of three biological replicates remained in normal growth conditions. Symptoms were observed every day to evaluate the chilling injury of different varieties.

Supplementary Figure 2 Physiological evaluation and principal component analysis (PCA) of seven pumpkin rootstock varieties under chilling treatment. **(A)** Malondialdehyde (MDA) content of the first true leaves of seven pumpkin varieties seedlings chilled at 4°C for 3 d. **(B)** PCA for the content of chlorophyll-a, chlorophyll-b, carotenoids and MDA and the REP and CII indices for seven pumpkin rootstock varieties. Three replicates for the pooled first true leaves and 9–12 individual seedlings per replicate were used for analysis. Asterisks indicate highly significant differences compared with chilling treatment at 0 d using the *t*-test for independent variables. Significance: ^∗∗∗^*p* < 0.001; ^∗∗^0.001 < *p* < 0.05; ^∗^0.05 < *p* < 0.01, ns = *p* > 0.01. Error bars indicate SD.

Supplementary Table 1 Oligonucleotides used for qRT-RCR verification.

Supplementary Table 2 Total variance explained in PCA.

Supplementary Table 3 Component matrix^*a*^ in PCA.

Supplementary Table 4 Distribution of principal components PC1 and PC2.

Supplementary Table 5 CmoDEGs in leaves of pumpkin seedlings under chilling stress.

Supplementary Table 6 CmoDEGs in roots of pumpkin seedlings under chilling stress.

Supplementary Table 7 Top 10 GO enrichment categories for 390 upregulated DEGs in pumpkin leaves under chilling stress.

Supplementary Table 8 KEGG enrichment of 390 upregulated DEGs in pumpkin leaves.

Supplementary Table 9 Top 10 GO enrichment categories of 620 upregulated DEGs in pumpkin roots under chilling stress.

Supplementary Table 10 KEGG enrichment of 620 upregulated CmoDEGs in roots.

Supplementary Table 11 Top 10 GO enrichment categories of 127 CmoDEGs in pumpkin leaves and roots under chilling stress.

Supplementary Table 12 KEGG enrichment of 127 CmoDEGs in leaves and roots of pumpkin seedlings under chilling stress.

Supplementary Table 13 Analysis of the expression of key genes involved in unsaturated fatty acid, β-oxidation and energy metabolism pathways in pumpkin rootstock leaves and roots.

Supplementary Table 14 The fatty acids content of the first leaves in pumpkin seedling under chilling and normal conditions.

## Abbreviations

12,13-EOTREO: 12,13(*S*)-epoxyoctade-catrienoic acid
13-HPOH: 13*S*-hydroperoxy-9*Z*,11*E*,15*Z*-octadecatrienoic acid
ACX: acyl-CoA oxidase
AOC: allene oxide cyclase
AOS: allene oxide synthase
AOX: alternative oxidase
*cis*-(+)-OPDA: *cis*-(+)-12-oxo-phytodienoic acid
DCMU: diuron
DEG: different gene expression
DOX: dioxygenase
FAD2: fatty acid desaturase 2
GH: glycosyl hydrolase
HPL: hydroperoxide lyase
JMT: jasmonic acid carboxyl methyltransferase
LOX: lipoxygenases
MFP: fatty acid beta-oxidation multifunctional protein
OPC8:0: 3-oxo-2-[(*Z*)pent-2 ′ -enyl]-cyclopentan-1-octanoic acid
OPR3: 12-oxophytodienoate reductase
PEP: phosphoenolpyruvate
PLA2: phospholipase A2 enzyme
PPCK: phosphoenolpyruvate carboxylase kinase
ROS: reactive oxygen species
VLCFAs: very-long-chain fatty acids.
