# Supplementary figures and images for: Transcriptomic and Physiological Analysis Reveal That α-Linolenic Acid Biosynthesis Responds to Early Chilling Tolerance in Pumpkin Rootstock Varieties
Source: Front Plant Sci. 2021 Apr 23;12:669565. doi: 10.3389/fpls.2021.669565 (PMC8104029; doi:10.3389/fpls.2021.669565)

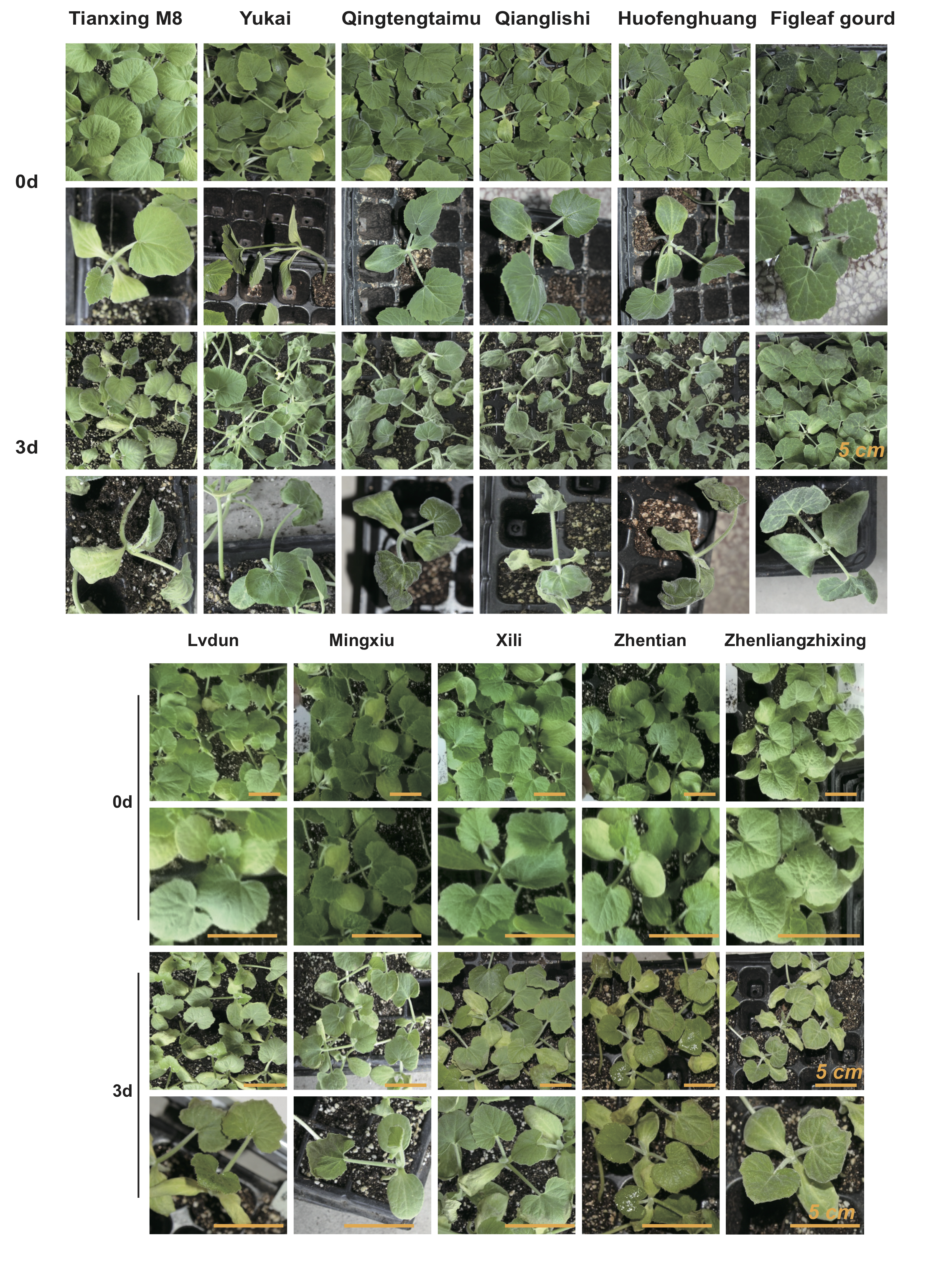

Supplement: Supplementary Figure 1 — Phenotypic changes in 14 pumpkin rootstocks after 5 d of chilling. Chilling treatments were applied to 100 seedlings at the two-true-leaf stage in each of three biological replicates, whereas another 100 seedlings in each of three biological replicates remained in normal growth conditions. Symptoms were observed every day to evaluate the chilling injury of different varieties. [file Image_1.TIF]

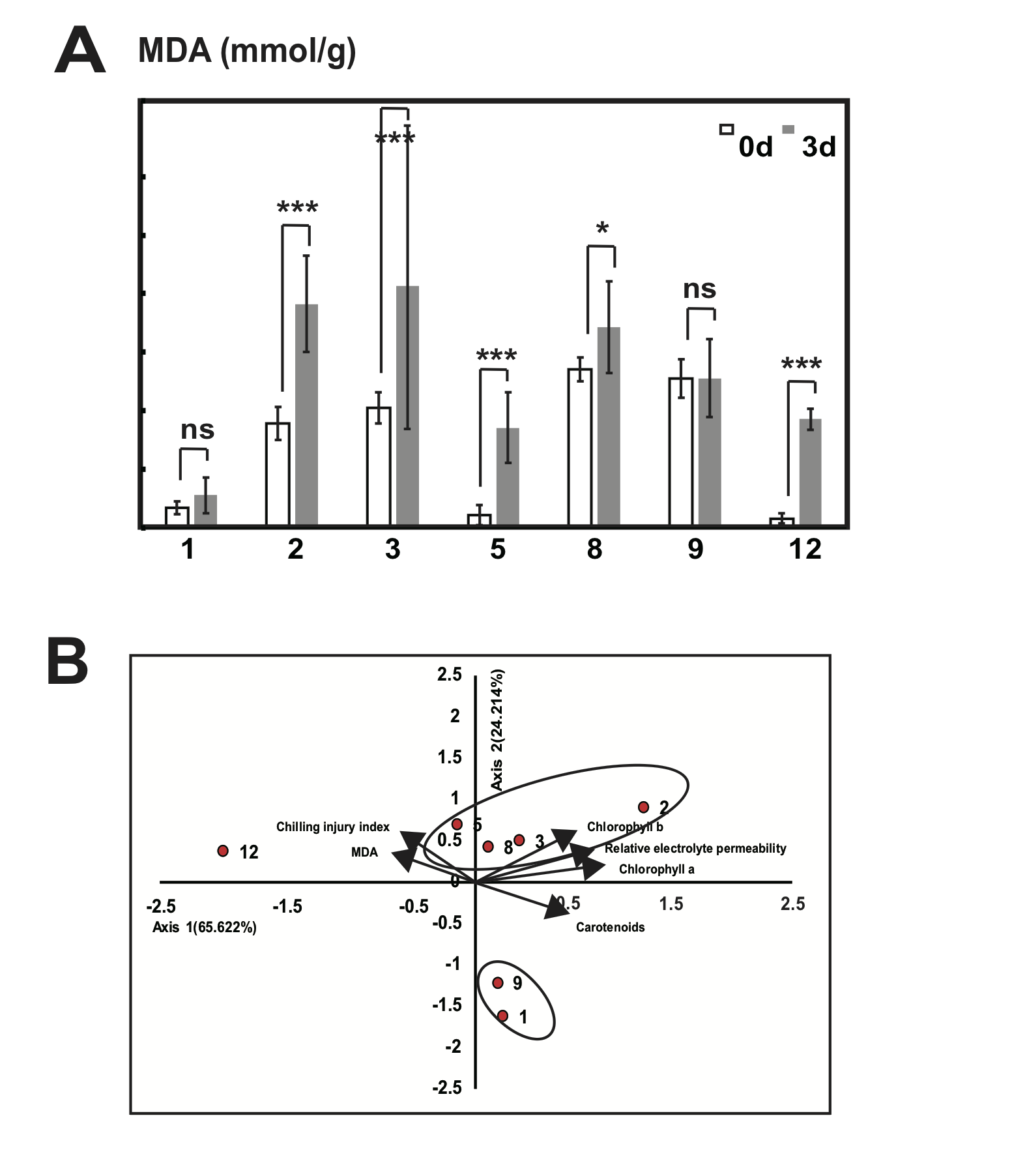

Supplement: Supplementary Figure 2 — Physiological evaluation and principal component analysis (PCA) of seven pumpkin rootstock varieties under chilling treatment. (A) Malondialdehyde (MDA) content of the first true leaves of seven pumpkin varieties seedlings chilled at 4°C for 3 d. (B) PCA for the content of chlorophyll-a, chlorophyll-b, carotenoids and MDA and the REP and CII indices for seven pumpkin rootstock varieties. Three replicates for the pooled first true leaves and 9–12 individual seedlings per replicate were used for analysis. Asterisks indicate highly significant differences compared with chilling treatment at 0 d using the t-test for independent variables. Significance: ∗∗∗p < 0.001; ∗∗0.001 < p < 0.05; ∗0.05 < p < 0.01, ns = p > 0.01. Error bars indicate SD. [file Image_2.TIF]
